# Dose-dependent changes in real-life affective well-being in healthy community-based individuals with mild to moderate childhood trauma exposure

**DOI:** 10.1186/s40479-023-00220-5

**Published:** 2023-04-20

**Authors:** Oksana Berhe, Carolin Moessnang, Markus Reichert, Ren Ma, Anna Höflich, Jonas Tesarz, Christine M. Heim, Ulrich Ebner-Priemer, Andreas Meyer-Lindenberg, Heike Tost

**Affiliations:** 1 Department of Psychiatry and Psychotherapy, Medical Faculty Mannheim, Central Institute of Mental Health, University of Heidelberg, Square J5, 68159 Mannheim, Germany; 2 Department of eHealth and Sports Analytics, Faculty of Sport Science, Ruhr-University Bochum, Bochum, Germany; 3 Department of Psychiatry and Psychotherapy, Medical University of Vienna, Vienna, Austria; 4 Department of Psychiatry and Psychotherapy, Karl Landsteiner University of Health Sciences, Tulln, Austria; 5 Department of General Internal Medicine and Psychosomatics, University Hospital Heidelberg, Heidelberg, Germany; 6 Institute of Medical Psychology, Charité – Universitätsmedizin Berlin, Corporate Member of Freie Universität Berlin, Humboldt-Universität zu Berlin, Berlin, Germany; 7 College of Health & Human Development, The Pennsylvania State University, University Park, PA USA; 8 mHealth Lab, Department of Applied Psychology, Institute of Sports and Sports Science, Karlsruhe Institute of Technology, Karlsruhe, Germany

**Keywords:** Community sample, Ecological momentary assessment, Childhood trauma exposure, Mental health risk

## Abstract

**Background:**

Childhood trauma exposures (CTEs) are frequent, well-established risk factor for the development of psychopathology. However, knowledge of the effects of CTEs in healthy individuals in a real life context, which is crucial for early detection and prevention of mental disorders, is incomplete. Here, we use ecological momentary assessment (EMA) to investigate CTE load-dependent changes in daily-life affective well-being and psychosocial risk profile in n = 351 healthy, clinically asymptomatic, adults from the community with mild to moderate CTE.

**Findings:**

EMA revealed significant CTE dose-dependent decreases in real-life affective valence (*p* = 0.007), energetic arousal (*p* = 0.032) and calmness (*p* = 0.044). Psychosocial questionnaires revealed a broad CTE-related psychosocial risk profile with dose-dependent increases in mental health risk-associated features (e.g., trait anxiety, maladaptive coping, loneliness, daily hassles; *p* values < 0.003) and a corresponding decrease in factors protective for mental health (e.g., life satisfaction, adaptive coping, optimism, social support; *p* values < 0.021). These results were not influenced by age, sex, socioeconomic status or education.

**Conclusions:**

Healthy community-based adults with mild to moderate CTE exhibit dose-dependent changes in well-being manifesting in decreases in affective valence, calmness and energy in real life settings, as well as a range of established psychosocial risk features associated with mental health risk. This indicates an approach to early detection, early intervention, and prevention of CTE-associated psychiatric disorders in this at-risk population, using ecological momentary interventions (EMI) in real life, which enhance established protective factors for mental health, such as green space exposure, or social support.

**Supplementary Information:**

The online version contains supplementary material available at 10.1186/s40479-023-00220-5.

## Introduction

Childhood trauma exposures (CTEs) such as physical, sexual, and emotional abuse and physical and emotional neglect are recognized environmental risk factors for the development of a wide range of adverse health outcomes across the lifespan [1–3]. For example, physical abuse, emotional abuse and neglect in childhood roughly double the likelihood of psychiatric disorders such as depression, anxiety disorders and substance abuse in adulthood [4], with a dose-dependent relationship between cumulative CTE exposure and severity of psychopathology [3, 5]. CTEs are further associated with a range of somatic conditions, including altered neuroendocrinological stress response, cardiovascular, inflammatory, and metabolic diseases and premature death [6]. Given that up to 30% of the adult population have experienced some form of CTEs [7], this represents a massive public health burden.

Research have linked CTEs with maladaptive personality traits, such as higher trait anxiety, neuroticism and lower optimism, self-efficacy and life satisfaction, suggesting a dispositional vulnerability [8–10]. This idea is further supported by ambulatory assessment studies reporting a CTE-related increase in daily negative affect [11], and stress experience [12]. At the same time, our knowledge of the effects of CTEs on mental health is still incomplete. First, our understanding of possible CTE-related affective changes in the general population is limited, especially when it comes to milder forms of adverse exposures and related subclinical changes in everyday life. Here, community-based studies and the availability of smartphone-based Ecological Momentary Assessment (EMA) in real life provide an opportunity for new insights [13, 14]. Second, most studies on CTEs have focused on patient populations [15]. These results need to be complemented by information about the nature and extent of subclinical changes in healthy individuals with CTE to define their risk for and resilience against developing a psychiatric disorder.

To fill this gap in knowledge, this study combined methods from psychology, epidemiology and EMA to investigate the psychosocial risk profile along with changes in daily-life affective well-being in healthy individuals with CTE from the general population. Based on our previous work with at-risk populations [13, 14, 16], we hypothesized that CTE, even in a clinically healthy group, would predict reduced momentary affective well-being in daily life and increased psychosocial risk for mental illness.

## Methods

### Study participants

We recruited in total 351 healthy young adults (mean age: 24.80 ± 6.54 years, 162 males) from local communities in the Rhine-Neckar metropolitan area in Germany for this study, thereby over-sampling for CTE to ensure an enrichment of CTE in the total sample. We provide further demographic details in Table 1 and sTable1. General exclusion criteria included the presence of a significant general medical disorder, neurological disorder, or a current or lifetime psychiatric disorder as determined by clinical interviews [17, 18]. None of the recruited subjects reported clinical psychiatric symptoms at the time of the study entry. Study participants gave written informed consent for a study protocol approved by the Medical Ethics Committee II of Heidelberg University.

### Questionnaire data acquisition and analysis

#### CTE

For retrospective assessment of CTEs we used the Childhood Trauma Screener (CTS), a validated instrument covering sexual, emotional and physical abuse and emotional and physical neglect [19]. Here, according to published cut-offs [19], and severity classifications [20] mild and moderate CTE can be defined as fulfilling one or two trauma subtypes respectively (see sMethods for details).

#### Sociodemographic and Psychosocial risk measures

Participants also completed a battery of sociodemographic and psychosocial assessments aiming at quantifying established psychosocial risk factors for mental health. This included trait anxiety, loneliness, self-efficacy, sense of coherence, optimism, mental well-being, life satisfaction, daily hassles, coping strategies and social support. We provide a full overview of the measures in sTable2. Because of the skewed distribution of CTS scores, we examined dose-dependent (linear) associations between cumulative CTE (CTS sum score) and questionnaire scores in SPSS (IBM, SPSS, version 25) using nonparametric Spearman rank correlation analyses corrected for age, sex, education, and SES (see sFigure1 and Table 1 for details).

### EMA and analysis

We assessed e-diary-ratings of the momentary social affective experience with study smartphones (Motorola Moto G, Motorola Mobility). EMA ratings were collected on 7 consecutive days in daily life with a flexible time- and location-dependent sampling schedule with an average of 12.51 ± 1.79 prompts per day, as previously described [14, 16, 21]. We assessed momentary well-being using a validated EMA short scale with good reliability and sensitivity [22]. The scale captures real-life *affective valence*, *calmness* and *energetic arousal* with two bipolar scales for each measure, presented as computerized visual analog scales with sliding locators (score range: 0 − 100). At an exploratory level, we also evaluated e-diary scales quantifying momentary social contact, social anhedonia, the appraisal of negative and positive events, and computed an established EMA measure of affective instability in daily life (mean square of successive differences, MSSD) from momentary affective valence scores [13, 14]. We nested within-subject e-diary assessments (level 1) within participants (level 2) and used multilevel models in SAS (version 9.4., SAS Institute Inc., Cary, NC, USA) to test for associations between cumulative CTE (CTS sum score) and EMA outcome measures. Since the distribution of the CTS sum variable was skewed (see sFigure 1) we log-transformed the variable using the natural logarithm. In addition, we added the level-1 predictors time of the day and time of the day squared, and age, sex, SES and years of education as level-2 covariates. Furthermore, we examined the distribution of the residuals of the models to rule out any bias due to the structure of the data (sFigure 2). We provide further methods details in the sMethods.

## Results

### Questionnaire data

**Fig. 1:**
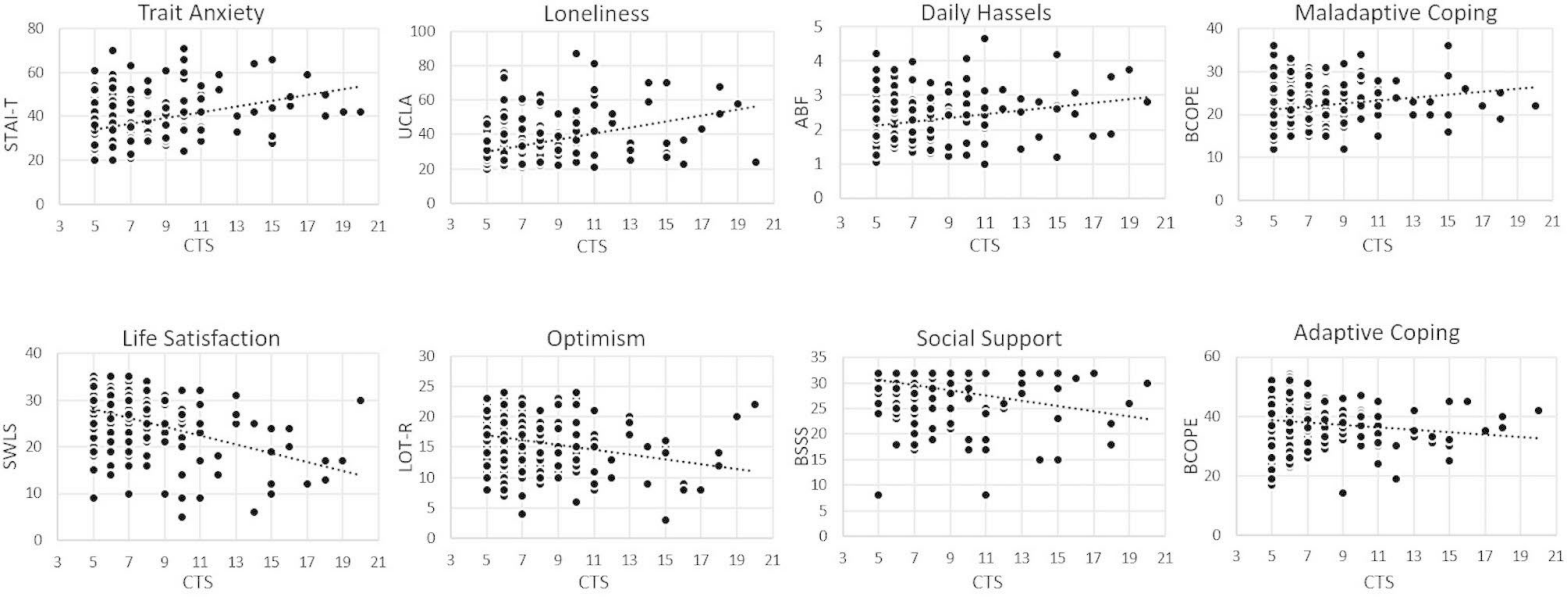
**Relationship between CTE load and psychosocial risk and protective factors for mental health.** Significant CTE dose-dependent increases in questionnaire measures capturing (upper row, from left to right) trait anxiety loneliness, daily hassles, and maladaptive coping (all *p* values < 0.003) and significant decrease in (bottom row, from left to right) life satisfaction, optimism, social support and adaptive coping (all *p* values < 0.021). X-axis: Childhood trauma screener (CTS) sum score; Y-axis: mean/sum values of questionnaire measures

Higher severity of CTE was significantly associated with older age, lower SES, and fewer years of education (*p* values < 0.026). After adjustment for age, sex, SES, and education, higher CTE load was significantly associated with higher scores for trait anxiety, loneliness, perceived daily hassles, and use of maladaptive coping strategies, as well as significantly lower scores for psychological well-being, life satisfaction, optimism, sense of coherence, self-efficacy, and perceived social support (*p* values < 0.021). We provide further details of the results in Table 1; Fig. 1. Details of the statistical relationships of the five CTS subdomains to the psychosocial measures are presented in sTable3 for exploratory purposes.

### Ecological momentary assessments

**Fig. 2:**
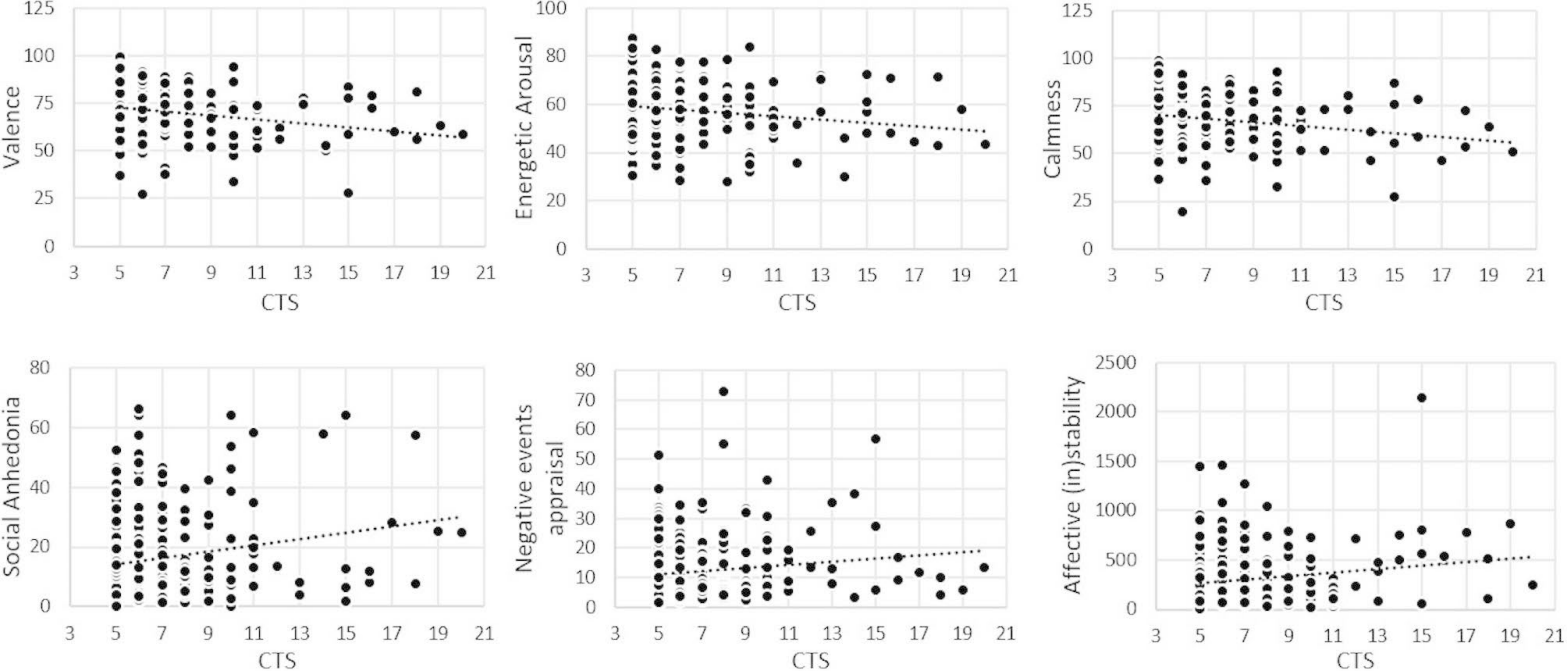
**Relationship between CTE load and real-life affective well-being.** Significant CTE dose-dependent decreases (upper row, from left to right) in real-life affective valence (*p* = 0.007), energetic arousal (*p* = 0.032,) and calmness (*p* = 0.044) in healthy community-based individuals with mild to moderate CTE. Absence of such associations in social-affective EMA measures reflecting evaluation of (bottom row, from left to right) social anhedonia (*p* > 0.23), appraisal of negative events (*p* > 0.98) and affective (in)stability (valence MSSD, *p* > 0.50). X-axis: Childhood trauma screener (CTS) sum score; Y-axis: mean values of EMA indices across 7 days of measurement

As hypothesized, higher trauma exposure during childhood was significantly associated with lower momentary affective valence in daily life in adulthood (*p* = 0.007). In addition, higher CTE related to lower momentary calmness and energetic arousal (p values < 0.044; see also Table 1; Fig. 2, and sTable4). Additional covariation for age, sex, SES, education and reported daily hassles did not change these results. In contrast, our exploratory analysis of EMA measures of affective instability, frequency and evaluation of momentary social contacts, and appraisal of positive and negative events in daily life revealed no significant associations with CTE burden. We provide further details of the EMA results in Table 1; Fig. 2, and sTable4.

## Discussion

In this study, we aimed to answer the question of whether there are dose-dependent changes in real-life affective well-being and psychosocial risk profile even in healthy asymptomatic individuals from the community with self-reported mild to moderate CTE. Consistent with our hypothesis, we observed a significant CTE dose-dependent decrease in affective valence in everyday life. In addition, we found a significant negative association between CTE load and momentary energy and calmness. Notably, we have previously observed a similar sensitivity of these measures in relation to other established psychiatric risk and resilience factors, including in individuals with subclinical symptoms [13] and healthy persons who benefited in well-being from social contact [14], physical activity [21] and exposure to urban green space [16]. Our study extends these data by showing that even clinically healthy, asymptomatic individuals from the community with milder forms of CTEs exhibit dose-dependent changes in real-life affective well-being as adults, and that these EMA scales are well suited to capture such risk-associated changes in naturalistic settings. Further longitudinal studies in healthy exposed individuals are needed to investigate the value of these risk markers of the development of CTE-related mental disorders in adulthood.

Based on the collected questionnaire measures, we further identified a psychosocial risk profile in our individuals with CTE consistent with our hypothesis. The profile consisted of a dose-dependent increase in several known psychosocial risk factors for mental health (e.g., trait anxiety, maladaptive coping, daily stress) and a corresponding decrease in factors known to reduce the odds of developing a psychiatric disorder (e.g., social support, adaptive coping, life satisfaction, optimism). Here, our data confirm and extend the existing knowledge by showing that, in addition to clinical populations with more pronounced CTEs [8, 23] similar dose-dependent psychosocial associations can be found in healthy community-based individuals with milder forms of CTEs. Furthermore, they indicate potential psychological mechanisms through which CTEs reduce well-being in this population.

These results should be evaluated in light of several study limitations. First, although our study includes a comparatively large sample, we cannot draw any causal conclusions because of the cross-sectional design used. Second, as in many other studies, our measure of CTE burden is based on a retrospective self-report instrument. Although such surveys may be biased, previous data suggest that CTEs are underreported rather than overreported on such measures and that they are not crucially influenced by current emotional states [24]. Third, the distribution of CTE load was skewed in our population-based healthy sample, which was to be expected. Thus, we took special precautions to obtain robust results by using nonparametric methods, log-transforming predictors, and examining the distribution of model residuals.

## Conclusion

In conclusion, we found that healthy, clinically asymptomatic individuals from the community with mild to moderate CTEs exhibit dose-dependent changes in well-being as adults. These changes manifest in a decrease in affective valence, calmness and energy in real life settings. We further identified a broad psychosocial risk profile, in which features detrimental to mental health accumulate. We hope that these data will contribute to a precision approach to early detection, early intervention, and prevention of CTE-associated psychiatric disorders in the general population. The risk-associated features established in this work (i.e., affective valence) could be targeted, for example, by EMI in real life, which enhance established protective factors for mental health, such as green space exposure, or social support [14, 16].

**Table 1:** Table 1

	Behavioral Sample (N = 351)			Associations with trauma load (CTS sum score)	
	M ± SD / count	min/max	n	*Spearman’s ρ /* *F value* ^*a*^	*p* value
***Demographic data*** ^***a***^					
Age (year)	24.80 ± 6.54	18.11/56.02	351	0.237	**< 0.001**
Sex (male/female)	162/189	N/A	351	0.068	0.201
Education (years)	13.27 ± 1.89	8/16	350	-0.119	**0.026**
Socioeconomic status (SES)	14.53 ± 3.16	6.20/21.00	350	-0.179	**0.001**
***Personality***					
NEO-FFI Openess (mean)	14.94 ± 4.89	1/24	347	0.017	0.751
NEO-FFI Agreeableness (mean)	17.67 ± 3.92	5/24	346	-0.113	**0.038**
NEO-FFI Conscientiousness (mean)	18.04 ± 3.63	6/24	346	-0.115	**0.033**
NEO-FFI Extraversion (mean)	15.08 ± 3.61	1/24	347	-0.217	**< 0.001**
NEO-FFI Neurotizismus (mean)	7.80 ± 4.63	1/24	348	0.175	**0.001**
***Risk Factors***					
**Early adversity (CTS, sum)**	6.29 ± 1.97	5/18	351	---	---
Trait anxiety (STAI-T, sum)	35.20 ± 8.57	20/71	349	0.321	**< 0.001**
Loneliness (UCLA, mean)	32.51 ± 10.51	20/87	346	0.396	**< 0.001**
Daily Stress (ABF)	2.21 ± 0.68	1.00/4.66	319	0.174	**0.003**
BCOPE-maladaptive coping (sum)	21.37 ± 4.22	12/36	343	0.164	**0.002**
***Protective Factors***					
Mental wellbeing (WHO-5, sum)	16.27 ± 4.09	2/25	346	-0.166	**0.002**
Satisfaction with life (SWLS)	27.06 ± 5.06	5/35	349	-0.310	**< 0.001**
Optimism (LOT-R optimism, sum)	16.52 ± 3.74	3/24	346	-0.279	**< 0.001**
Sense of coherence (SOC, sum)	148.71 ± 19.68	72/197	345	-0.342	**< 0.001**
Self-efficacy (SWE, sum)	30.38 ± 4.27	17/40	349	-0.147	**0.007**
Perceived social support (BSSS, sum)	29.83 ± 3.61	8/32	350	-0.312	**< 0.001**
BCOPE-adaptive coping (sum)	37.90 ± 6.37	14/54	343	-0.120	**0.021**
***EMA*** ^***b***^					
Valence (MDBF)^c^	75.08 ± 11.21	27.16/99.14	347	7.43	**0.007**
Energetic arousal (MDBF)^c^	58.80 ± 11.14	27.96/87.45	347	4.65	**0.032**
Calmness (MDBF)^c^	69.55 ± 11.55	19.73/98.59	347	4.09	**0.044**
Alone (alone/not alone)^d^	33/84	NA	347	0.83	0.363
Don’t like the company^c^	10.54 ± 9.74	0.24/69.12	347	1.40	0.237
Rather be alone^c^	14.96 ± 13.58	0.17/66.17	347	2.48	0.117
Positive event appraisal^c^	23.64 ± 13.84	1.5/75.71	347	1.86	0.174
Negative event appraisal^c^	11.29 ± 7.97	0.67/55.00	347	0.00	0.982
Affective (in)stability (valence MSSD)	275.05 ± 211.71	1.07/1448.43	347	0.44	0.508
E-diary prompts per day	12.51 ± 1.79	7/20	347	-0.084	0.118
E-diary Compliance (%)^***e***^	81.59 ± 14.26	32.93/100	347	-0.088	0.105

**CTE dose-dependent changes in real-life well-being and psychosocial metrics, well-known as risk and protective factors for mental well-being.** For details on the psychosocial questionnaires including acronyms, see *Supplemental Table S2*. Abbreviations: M = mean, SD = standard deviation, n = number of participants with available data, MSSD valence = mean square of successive differences of valence ratings.

^a^Spearman’s correlation, partial spearman’s correlation controlling for sex, age, SES, education; F-values from multilevel analysis (controlling for time of the day and time of the day-squared, level 1, and for sex, age, SES, education, and daily hassles, level 2, see Methods section and sMethods for details).

^***b***^Aggregation of EMA indices within and between subjects for use for sample description only;

^c^mean/day/participant,

^d^number of prompts participants reported being alone out of all prompts across 7 days of measurement, mean;

^***e***^Percent of answered prompts.

## Electronic supplementary material

Below is the link to the electronic supplementary material.

Supplementary Material 1

## Data Availability

The data supporting the findings of this study are available upon request from the corresponding author HT. The data are not publicly available due to sensitive information that could compromise research participant privacy/consent.

## Abbreviations

ABF: Alltagsbelastungsfragebogen
CTE: Childhood Trauma Exposure
BSSS: Berlin Social Support Scale
CSSS: Chronic Stress Screening Scale
CTS: Childhood Trauma Screener
CTQ: Childhood Trauma Questionnaire
EMA: Ecological Momentary Assessment
EA: emotional abuse
EMI: ecological momentary intervention
EN: emotional neglect
GSES: General Self-Efficacy Scale
LOT-R: Revised Life Orientation Test
M: mean
MLA: multilevel analysis
MSSD: mean square of successive differences
n: number of participants with available data
PA: physical abuse
PN: physical neglect
SD: standard deviation
SES: socioeconomic status
SA: sexual abuse
STAI-T: State-Trait Anxiety Inventory
SOC: Sense of Coherence Scale
UCLA: UCLA Loneliness Scale
WHO: World health organization
SWLS: Satisfaction with Life Scale
