# Supplementary material for: Dose-dependent changes in real-life affective well-being in healthy community-based individuals with mild to moderate childhood trauma exposure
Source: Borderline Personal Disord Emot Dysregul. 2023 Apr 20;10:14. doi: 10.1186/s40479-023-00220-5 (PMC10116660; doi:10.1186/s40479-023-00220-5)
Supplement: Supplementary file 1 — Supplementary Material 1 [file 40479_2023_220_MOESM1_ESM.docx]

**Supplemental information**

**Dose-dependent changes** **in real-life affective well-being in healthy community-based individuals with mild to moderate childhood trauma exposure**

**Authors**

Oksana Berhe^1^, Carolin Moessnang^1^, Markus Reichert^1,2^, Ren Ma^1^, Anna Höflich^1,3,4^, Jonas Tesarz^5^, Christine M. Heim^6,7^, Ulrich Ebner-Priemer^1,8^, Andreas Meyer-Lindenberg^1^, Heike Tost^1^

^1^Department of Psychiatry and Psychotherapy, Central Institute of Mental Health, Medical Faculty Mannheim, University of Heidelberg, Mannheim, Germany

^2^Department of eHealth and Sports Analytics, Faculty of Sport Science, Ruhr-University Bochum, Bochum, Germany

^3^Department of Psychiatry and Psychotherapy, Medical University of Vienna, Vienna, Austria

^4^Department of Psychiatry and Psychotherapy, Karl Landsteiner University of Health Sciences, Tulln, Austria

^5^Department of General Internal Medicine and Psychosomatics, University Hospital Heidelberg, Heidelberg, Germany

^6^Charité – Universitätsmedizin Berlin, Corporate Member of Freie Universität Berlin, and Humboldt-Universität zu Berlin, Institute of Medical Psychology, Berlin, Germany

^7^College of Health & Human Development, The Pennsylvania State University, University Park, PA, USA

^8^mHealth Lab, Department of Applied Psychology, Institute of Sports and Sports Science, Karlsruhe Institute of Technology, Karlsruhe, Germany

^#^These authors contributed equally

**Supplemental Methods**

**Participants.** The initial sample of young adults (N=310, 24.02 ±2.83 [mean ± SD]) was recruited via the local population registry from local communities in the Rhine-Neckar metropolitan area, taking into account stratification of the population according to age, sex and nationality. The study population was enriched for the presence of CTE. Additional 41 (38.26 ±10.12 [mean ± SD]) individuals were recruited from August 2019 to October 2020 via advertisement from the same neighborhoods using the same enrollment criteria over-sampling for CTE. The total sample size used in the current analysis was N=351 (24.80 ± 6.54 [mean ± SD]) individuals (See Supplemental Table S1 for sample overview).

**Questionnaire data**

*CTE***.** To assess childhood trauma exposure we used Childhood trauma screener (CTS(1)). The CTS is a short self-report retrospective inventory with a total of 5 items developed from the German version of the “Childhood Trauma Questionnaire” (CTQ, 28 items) (2). Based on a large population sample on each of the 5 subscales of the CTQ was selected the item that best represented the dimension in terms of selectivity, explanation of variance and practicability. With high internal consistency (α=0.757), CTS is a reliable and economical screener for recording of adverse childhood experiences and recommended in diagnostic processes (1, 2). Thus, CTS is a 5-point Likert scale ranging from “Never true” to “Very often true”. The CTS contains five subscales, which measure three types of abuse and two types of neglect: namely emotional abuse (EA), physical abuse (PA), sexual abuse (SA), emotional neglect (EN), and physical neglect (PN). The score for each scale is a sum of scores of specific items, and the total score of the CTS is a sum of scores on all scales (range: 5 - 25) (1). Based on the established cut-off scores set for each type of trauma (EA: >2; PA: >2; SA: >1; EN: >3; PN: >3), presence of specific trauma types (yes/no) can be determined (1). In addition, trauma load has been defined based on the number of trauma types fulfilled (no CTE: none of the trauma subcategories fulfilled; mild CTE: one trauma subcategory fulfilled; moderate CTE: >2 trauma subcategories fulfilled) (3).

*Sociodemographic and psychosocial risk measures***.** All participants further completed a comprehensive battery of sociodemographic measures and psychosocial assessment comprising an established multidimensional aggregated index of socioeconomic status (SES (4)), personality (5), trait anxiety (6), loneliness (7), perceived daily hassles (8), self-efficacy (9), sense of coherence (10), optimism (11), perceived wellbeing (12), satisfaction with life (13), coping strategies (14), perceived social support (15). The detailed overview of the self-reported battery is published elsewhere and given in the Supplemental Table S2 (16).

**Ecological momentary assessments**

*Hardware.* The 7-days EMA protocol included smartphone-based e-diary assessments (Motorola Moto G, Motorola Mobility), GPS-based location tracking, as well as accelerometry (movisens Move-II or movisens Move-III, movisens GmbH). We employed a flexible time and location-based sampling scheme for e-diary assessments. That is, each participants received between 9-23 prompts/day between 7:30 and 22:30 (fixed prompts at 8:00 and 22:20), with a minimum interval of 40 min and a maximum interval of 100min (17-19). Assessments were done via a Smartphone App using the software movisensXS, version 0.6.3658 (movisens GmbH, <https://xs.movisens.com>).

*Measures*. As our main outcome variable, real-life affective well-being (indexed by valence, calmness and energetic arousal), we used a well-known short scale for EMA with established psychometric properties (20). Momentary valence was measured with the two bipolar items “content” to “discontent” (German translation: “zufrieden” – “unzufrieden”) and “unwell” to “well” (German translation: “unwohl” – “wohl”); momentary calmness with the two bipolar items “tense” to “relaxed” and “agitated” to “calm”, and momentary energetic arousal with the two bipolar items “without energy” – “full of energy” and “tired” – “awake”.

All above items presented as computerized visual analogue scales with sliding locators (score range of 0 to 100).’’

In addition we acquired, at each prompt, e-diaries quantifying momentary social contact, social (an)hedonia (19, 21) and appraisals of positive and negative events (22). We assessed momentary social contact with a dichotomous item asking subjects whether they are currently alone or in the company of others. In the case if in company, we additionally asked the participants to rate, via visual analogue scales (score range of 0 to 100), the liking of the company (*“When you think about the people who surround you right now, to what extent is the following statement true? I don't like these people*!*”*), and the degree to which the subjects would prefer to be alone (*“When you think about the people who surround you right now, to what extent is the following statement true? I’d rather be alone!*“) (19, 21). We assessed positive and negative event appraisal with two coverage items *(“Have you experienced one or more negative events since the last query? How intense was the most important negative event? Have you experienced one or more pleasant events since the last query? How intense was the most important pleasant event?”)* that the participants rated using visual analogue scales with score ranges of 0 – 100 (‘no event’=0 to very intense’ =100).

*Multilevel modeling of e-diary assessments.* To test our main hypothesis, we estimated the effect of childhood trauma exposure (indexed by CTS) on affective valence, calmness and energetic arousal (3 models) by conducting random-intercept multilevel model analysis in SAS (version 9.4., SAS Institute Inc., Cary, NC, USA), thereby nesting e-diary assessments (level 1) within participants (level 2) (23). Besides our main predictor CTS (log-transformed), we added the level-1 predictors time of the day and time of the day squared (transformed to the daily study start time at 7:30 AM) to the model to control for known time-of-day effects on affective valence (17, 24). Furthermore, we included age, sex, SES and years of education as level-2 covariates. Following established procedures (23), we incorporated random effects for both the intercept and eacsh predictor and subsequently deleted non-significant random effects. Equation S1 details the full model below using a single equation representation.

*Equation (S1):*

$${Y(\boldsymbol{affective valence})}_{ij}=\beta_{00}+\beta_{01}*\boldsymbol{CTS}_{j}+\beta_{02}*{age}_{j}+\beta_{03}*{sex}_{j}+\beta_{04}*{SES}_{j}+\beta_{05}*{educ}_{j}+\beta_{10}*{time of day}_{ij}+ \beta_{20}*{time of day}_{ij}^{2} {+ u}_{0j}{+ u}_{1j}*{time of day}_{ij} +r_{ij}$$

Y*_ij_* represents the level of affective valence in person *j* at time *i*. Within-subject effects are modeled on level 1, represented by each participant’s (subscript *j*) value entries for every prompt (subscript *i*). Beta coefficients denote the intercept, the effect of our main predictor CTS (log-transformed), of age, sex, SES, and education, and the effects of the level-1 covariates (time of the day, time of the day squared). Random effects, i.e., individual variation around the sample mean, are represented by u*_0j_* for the intercept and u*_1j_* – for time of the day. In the same vein, we set up two other separate models with calmness and energetic arousal as an outcome variable. For exploratory analysis, we further computed analogous models for e-diary outcomes reflecting additional daily-life processes (see above, and *Methods* and *Table 1* in the main text).

**Supplemental Results**

**Questionnaire data.** As expected, the distribution of the CTS score in our sample was skewed to the left with the M= 6.29±1.97 (with a range of 5-25: ‘5’ being the minimum value for selecting ‘never true’ for all 5 items), suggesting for a predominantly non-traumatized sample (sFigure 1). A substantial proportion of individuals in our sample (N=259) reported no-to mild CTE; 93 participants reported moderate level of CTE (based on the CTS classification, see sMethods for details)(1, 3).

**Ecological momentary assessments.** Two datasets had to be excluded from the subsequent analysis due to low compliance (<30) (see Table 1 in the main text and Table S3 for more details). On average, participants responded to 12.51 prompts per day (SD = 1.79; range = 7/20), resulting in a high compliance rate of 81.59%.

**Supplemental Table S1: Sample overview**

|  | **Total Sample (N=351)** | | | **PEZ basic Cohort (N=310)** | | | **Enriched Sample (N=41)** | | |
| --- | --- | --- | --- | --- | --- | --- | --- | --- | --- |
|  | M ± SD / count | min/max | n | M ± SD / count | min/max | n | M ± SD / count | min/max | n |
| ***Demographic data*** |  |  |  |  |  |  |  |  |  |
| Age (year) | 24.80 ±6.54 | 18.11/56.02 | 351 | 24.02 ±2.83 | 18.11/28.46 | 310 | 38.26 ±10.12 | 20.14/55.02 | 41 |
| Sex (male/female) | 162/189 | N/A | 351 | 141/169 | N/A | 310 | 21/20 | N/A | 41 |
| Education (years) | 13.27 ± 1.89 | 8/16 | 350 | 13.07 ± 1.85 | 8/16 | 310 | 14.84 ±2.96 | 7.20/19.20 | 40 |
| Socioeconomic status (SES) | 14.53± 3.16 | 6.20/21.00 | 350 | 14.49 ± 3.18 | 6.20/21.00 | 310 | 14.72 ±1.60 | 11.00/16.00 | 40 |

**Supplemental Table S2: Inventory measures**

| **Name** | **Outcome measure(s)** | **Details** |
| --- | --- | --- |
| Socioeconomic status assessment of the German Health Update 2009 (GEDA)(4) | Socioeconomic status | Multidimensional aggregated index of status-constituting dimensions (education, occupation, income) |
| NEO Five-Factor Inventory(5) | Measure of the five domains of personality (Neuroticism, Extraversion, Openness, Agreeableness, and Conscientiousness) | 60 items (12 per trait). 5-point Likert scale |
| Childhood Trauma Screener (CTS)(1) | Retrospective self-rating of adverse childhood experiences | 5 items, 5-point Likert scales |
| State-Trait Anxiety Inventory (STAI-T)(6) | Trait anxiety | 20 items, 4-point Likert scales |
| UCLA Loneliness Scale (UCLA)(7) | Perceived loneliness | 20 items, 4-point Likert scales |
| Alltagsbelastungsfragebogen (ABF)(8) | Daily hassles | 58 items, 8-point Likert scales |
| Brief-COPE(14) | Maladaptive coping strategies  Adaptive coping strategies | 28 items, 4-point Likert scales |
| WHO-5 Well-Being Index(12) | Perceived current mental well-being | 5 items, 6-point Likert scales |
| Satisfaction with Life Scale (SWLS)(13) | Global cognitive judgments of satisfaction with life | 5 items, 7-point Likert scales |
| Revised Life Orientation Test (LOT-R)(11) | Dispositional optimism | 10 items, 5-point Likert scales |
| Sense of Coherence Scale (SOC-29)(10) | Dispositional sense of coherence | 29 items, 5-point Likert scales |
| General Self-Efficacy Scale (GSES)(9) | Generalized belief in the own ability to control environmental challenges | 10 items, 4-point Likert scales |
| Berlin Social Support Scale (BSSS)(15) | Perceived social support | 8 items, 4-point Likert scales |

**Supplemental Table S3:** Relationships of the five CTS subdomains to the psychosocial measures

|  | **Relation to CTS: emotional neglect** | | **Relation to CTS:**  **physical abuse** | | **Relation to CTS:**  **emotional abuse** | | **Relation to CTS:**  **sexual abuse** | | **Relation to CTS:**  **physical neglect** | |
| --- | --- | --- | --- | --- | --- | --- | --- | --- | --- | --- |
|  | **Spearman's ρ** | *p* value | **Spearman's ρ** | *p* value | **Spearman's ρ** | *p* value | **Spearman's ρ** | *p* value | **Spearman's ρ** | *p* value |
| ***Demographic data^a^*** |  |  |  |  |  |  |  |  |  |  |
| Age (year) | **0.158** | **0.003** | **0.182** | **0.001** | 0.072 | 0.178 | **0.121** | **0.023** | **0.165** | **0.002** |
| Sex (male/female) | **0.171** | **0.001** | 0.055 | 0.303 | -0.057 | 0.289 | **-0.115** | **0.032** | 0.048 | 0.367 |
| Education (years) | 0.052 | 0.336 | 0.091 | 0.090 | 0.040 | 0.452 | 0.085 | 0.112 | 0.009 | 0.870 |
| Socioeconomic status (SES) | **-0.169** | **0.002** | -0.087 | 0.104 | **-0.134** | **0.012** | -0.81 | 0.131 | **-0.156** | **0.004** |
| ***Personality^b^*** |  |  |  |  |  |  |  |  |  |  |
| NEO-FFI Openess (mean) | 0.026 | 0.636 | 0.013 | 0.807 | 0.074 | 0.178 | 0.076 | 0.165 | -0.049 | 0.368 |
| NEO-FFI Agreeableness (mean) | **-0.153** | **0.005** | 0.004 | 0.943 | **-0.141** | **0.010** | -0.045 | 0.407 | -0.011 | 0.840 |
| NEO-FFI Conscientiousness (mean) | -0.063 | 0.246 | **-0.121** | **0.026** | -0.075 | 0.170 | -0.106 | 0.052 | -0.052 | 0.344 |
| NEO-FFI Extraversion (mean) | **-0.277** | **<0.001** | 0.065 | 0.234 | -0.080 | 0.143 | -0.073 | 0.184 | **-0.117** | **0.032** |
| NEO-FFI Neurotizismus (mean) | **0.151** | **0.006** | 0.006 | 0.918 | **0.210** | **<0.001** | 0.041 | 0.455 | 0.060 | 0.270 |
|  |  |  |  |  |  |  |  |  |  |  |
| ***Risk Factors*** |  |  |  |  |  |  |  |  |  |  |
| Trait anxiety (STAI-T, sum) | **0.276** | **<0.001** | 0.042 | 0.437 | **0.251** | **<0.001** | 0.081 | 0.129 | **0.156** | **0.003** |
| Loneliness (UCLA, mean) | **0.374** | **<0.001** | **0.120** | **0.025** | **0.218** | **<0.001** | 0.065 | 0.226 | **0.278** | **<0.001** |
| Daily Stress (ABF) | 0.096 | 0.099 | 0.036 | 0.533 | **0.175** | **0.002** | 0.044 | 0.454 | -0.012 | 0.843 |
| BCOPE-maladaptive coping | **0.126** | **0.019** | 0.048 | 0.378 | **0.208** | **<0.001** | 0.062 | 0.252 | **0.105** | **0.052** |
|  |  |  |  |  |  |  |  |  |  |  |
| ***Protective Factors*** |  |  |  |  |  |  |  |  |  |  |
| Mental wellbeing (WHO-5, sum) | **-0.105** | **0.050** | -0.019 | 0.729 | **-0.183** | **0.001** | -0.011 | 0.841 | -0.046 | 0.397 |
| Satisfaction with life (SWLS) | **-0.297** | **<0.001** | **-0.177** | **0.001** | **-0.208** | **<0.001** | **-0.139** | **0.010** | **-0.245** | **<0.001** |
| Optimism (LOT-R, optimism sum) | **-0.243** | **<0.001** | -0.038 | 0.487 | **-0.161** | **0.003** | -0.077 | 0.154 | **-0.146** | **0.007** |
| Sense of coherence (SOC, sum) | **-0.261** | **<0.001** | 0.009 | 0.862 | **-0.270** | **<0.001** | -0.007 | 0.897 | **-0.203** | **<0.001** |
| Self-efficacy (SWE, sum) | **-0.109** | **0.043** | 0.037 | 0.491 | **0.098** | **0.067** | -0.031 | 0.558 | **-0.107** | **0.046** |
| Perceived social support (BSSS, sum) | **-0.336** | **<0.001** | **-0.143** | **0.007** | **-0.224** | **<0.001** | 0.012 | 0.828 | **-0.230** | **<0.001** |
| BCOPE-adaptive coping | **-0.179** | **0.001** | **-0.120** | **0.026** | -0.048 | 0.379 | -0.046 | 0.392 | **-0.177** | **0.001** |
|  |  |  |  |  |  |  |  |  |  |  |

For details on the psychosocial questionnaires including acronyms, see *Supplemental Table S2*.

^a^ Spearman’s correlation;

^b^ Partial spearman’s correlation controlling for sex, age, SES, education;

**Supplemental Table S4:** Multilevel analysis results, main models

| **Dependent variable** | **Predictor** |  | | | |
| --- | --- | --- | --- | --- | --- |
|  | ***Fixed effects*** | **Beta coefficient** | **Standard Error** | ***T value* (df)** | ***P value*** |
|  |  |  |  |  |  |
| ***Valence***  (0-100) | Intercept | 93.12 | 6.74 | 13.81 (337) | <0.001 |
|  | Time (hours) | 0.61 | 0.08 | 7.19(11E3) | <0.001 |
|  | Time-squared (hours^2^) | -0.02 | 0.00 | -4.09(24E3) | <0.001 |
|  | Sex: male | -3.34 | 1.25 | -2.67(337) | 0.008 |
|  | Age | 0.11 | 0.12 | 0.95(336) | 0.345 |
|  | SES | -0.23 | 0.21 | -1.11(336) | 0.269 |
|  | Education | -0.07 | 0.37 | -0.19(336) | 0.848 |
|  | **Childhood trauma exposure (CTS)** | **-22.44** | **5.81** | **-3.86(337)** | **<0.001** |
|  |  |  |  |  |  |
|  |  |  |  |  |  |
| **Energetic Arousal**  (0-100) | Intercept | 51.24 | 8.50 | 6.03(335) | <0.001 |
|  | Time (hours) | 5.35 | 0.13 | 41.86(2661) | <0.001 |
|  | Time-squared (hours^2^) | -0.36 | 0.01 | -54.31(24E3) | <0.001 |
|  | Sex: male | -2.06 | 1.58 | -1.31(335) | 0.191 |
|  | Age | 0.64 | 0.14 | 4.63(333) | <0.001 |
|  | SES | -0.32 | 0.26 | -1.24(334) | 0.217 |
|  | Education | 0.26 | 0.47 | 0.55(334) | 0.584 |
|  | **Childhood trauma exposure (CTS)** | **-21.20** | **7.32** | **-2.90(334)** | **0.004** |
|  |  |  |  |  |  |
|  |  |  |  |  |  |
| **Calmness**  (0-100) | Intercept | 91.38 | 6.91 | 13.23(340) | <0.001 |
|  | Time (hours) | -0.61 | 0.09 | -6.94(11E3) | <0.001 |
|  | Time-squared (hours^2^) | 0.06 | 0.01 | 10.49(24E3) | <0.001 |
|  | Sex: male | -1.34 | 1.28 | -1.05(339) | 0.296 |
|  | Age | 0.10 | 0.11 | 0.87(338) | 0.383 |
|  | SES | -0.16 | 0.21 | -0.75(338) | 0.452 |
|  | Education | -0.28 | 0.38 | -0.74(338) | 0.462 |
|  | **Childhood trauma exposure (CTS)** | **-20.54** | **5.95** | **-3.45(339)** | **0.006** |
|  |  |  |  |  |  |

Abbreviations: SES = socioeconomic status, df = degree of freedom, All reported *p* values for beta coefficients are two-sided and derived from the t-statistics of the multilevel model.

**Supplemental Table S4 Continuation:** Multilevel analysis results, exploratory models

| **Dependent variable** | **Predictor** | **Beta coefficient** | **Standard error** | ***T value* (df)** |  |
| --- | --- | --- | --- | --- | --- |
|  | ***Fixed effects*** |  |  |  | ***P value*** |
|  |  |  |  |  |  |
| ***Model 1:***  Affective Instability | Intercept | 714.06 | 149.15 | 4.79(331) | <0.001 |
|  | Time (hours) | 7.71 | 4.51 | 1.71(19E3) | 0.087 |
|  | Time-squared (hours^2^) | -0.32 | 0.27 | -1.23(22E3) | 0.221 |
|  | Sex: male | -29.26 | 21.82 | -1.34(322) | 0.181 |
|  | Age | -0.47 | 1.92 | -0.24 (318) | 0.807 |
|  | SES | 0.72 | 3.56 | 0.20(321) | 0.840 |
|  | Education | -1.99 | 6.44 | -0.31(320) | 0.758 |
|  | Valence | -5.22 | 0.98 | -5.31(325) | <0.001 |
|  | **Childhood trauma exposure (CTS)** | -36.81 | 103.13 | -0.36(325) | 0.721 |
|  |  |  |  |  |  |
|  |  |  |  |  |  |
| ***Model 2:***  Don’t like the company  (0-100) | Intercept | -0.38 | 6.50 | -0.06(297) | 0.953 |
|  | Time (hours) | 0.15 | 0.13 | 1.15(8626) | 0.5248 |
|  | Time-squared (hours^2^) | -0.04 | 0.01 | -4.66(12E3) | <0.001 |
|  | Sex: male | 2.21 | 1.19 | 1.86(295) | 0.064 |
|  | Age | -0.09 | 0.11 | -0.83(309) | 0.405 |
|  | SES | 0.15 | 0.20 | 0.78(294) | 0.433 |
|  | Education | 0.19 | 0.36 | 0.53(298) | 0.597 |
|  | **Childhood trauma exposure (CTS)** | 8.79 | 5.57 | 1.58(297) | 0.116 |
|  |  |  |  |  |  |
|  |  |  |  |  |  |
| ***Model 3:***  Rather be alone  (0-100) | Intercept | -7.54 | 8.93 | -0.84(319) | 0.399 |
|  | Time (hours) | 0.37 | 0.16 | 2.27(7795) | 0.023 |
|  | Time-squared (hours^2^) | -0.05 | 0.01 | -5.32(12E3) | <0.001 |
|  | Sex: male | 2.15 | 1.63 | 1.31(317) | 0.190 |
|  | Age | 0.08 | 0.15 | 0.51(332) | 0.608 |
|  | SES | 0.12 | 0.27 | 0.44(317) | 0.662 |
|  | Education | 0.38 | 0.49 | 0.77(320) | 0.441 |
|  | **Childhood trauma exposure (CTS)** | 15.24 | 7.66 | 1.99(319) | 0.047 |
|  |  |  |  |  |  |
|  |  |  |  |  |  |
| ***Model 4:***  Positive Event Appraisal  (0-100) | Intercept | 19.24 | 7.61 | 2.53(334) | 0.012 |
|  | Time (hours) | 1.40 | 0.15 | 9.39(13E3) | <0.001 |
|  | Time-squared (hours^2^) | -0.05 | 0.01 | -4.97(24E3) | <0.001 |
|  | Sex: male | -1.90 | 1.41 | -1.34(333) | 0.179 |
|  | Age | 0.01 | 0.13 | 0.02(330) | 0.988 |
|  | SES | 0.21 | 0.23 | 0.90(334) | 0.367 |
|  | Education | 0.15 | 0.42 | 0.36(331) | 0.716 |
|  | **Childhood trauma exposure (CTS)** | -6.65 | 6.56 | -1.01(334) | 0.312 |
|  |  |  |  |  |  |
|  |  |  |  |  |  |
| ***Model 5:***  Negative Event Appraisal  (0-100) | Intercept | 4.17 | 4.39 | 0.95(319) | 0.343 |
|  | Time (hours) | 0.03 | 0.10 | 0.27(16E3) | 0.783 |
|  | Time-squared (hours^2^) | -0.01 | 0.01 | -0.70(24E3) | 0.485 |
|  | Sex: male | -0.30 | 0.81 | -0.36(317) | 0.716 |
|  | Age | 0.11 | 0.07 | 1.58(313) | 0.115 |
|  | SES | 0.07 | 0.13 | -0.53(317) | 0.600 |
|  | Education | -0.10 | 0.24 | -0.40(315) | 0.688 |
|  | **Childhood trauma exposure (CTS)** | 6.29 | 3.78 | 1.66(318) | 0.098 |
|  |  |  |  |  |  |
|  |  |  |  |  |  |
| ***Model 6:***  Being Alone  (1/0) | Intercept | 1.52 | 0.12 | 2.14(314.2) | <0.001 |
|  | Time (hours) | 0.03 | 0.00 | 10.36(10124) | <0.001 |
|  | Time-squared (hours^2^) | -0.00 | 0.00 | -7.33(23670) | <0.001 |
|  | Sex: male | -0.03 | 0.02 | -1.36(313.2) | 0.176 |
|  | Age | 0.00 | 0.00 | -1.64(310) | 0.102 |
|  | SES | 0.00 | 0.00 | 0.23(311.8) | 0.815 |
|  | Education | 0.01 | 0.01 | 1.24(313.6) | 0.218 |
|  | **Childhood trauma exposure (CTS)** | -0.15 | 0.11 | -1.44(313.8) | 0.151 |
|  |  |  |  |  |  |

Abbreviations: SES = socioeconomic status, df = degree of freedom, All reported *p* values for beta coefficients are two-sided and derived from the t-statistics of the multilevel model.

**
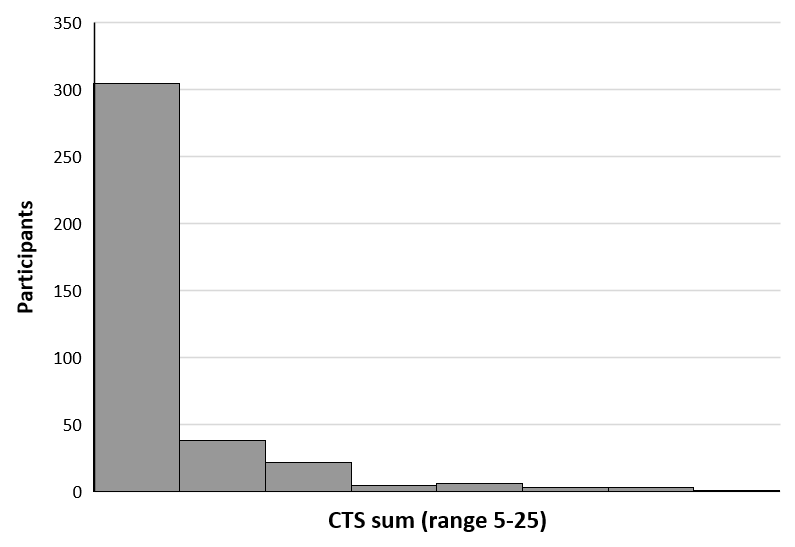
Supplemental Figure S1:** Distribution of childhood trauma exposure measured by CTS.

The histogram depicts the distribution (y-axis - frequency) of childhood trauma exposure (x-axis - CTS sum), indicating the skewed distribution of the measure.

**Supplemental Figure S2:** Distribution of level-1 residuals of the main hypothesis-testing multi-level models (valence, energetic, and calmness) (see Table S3)

**
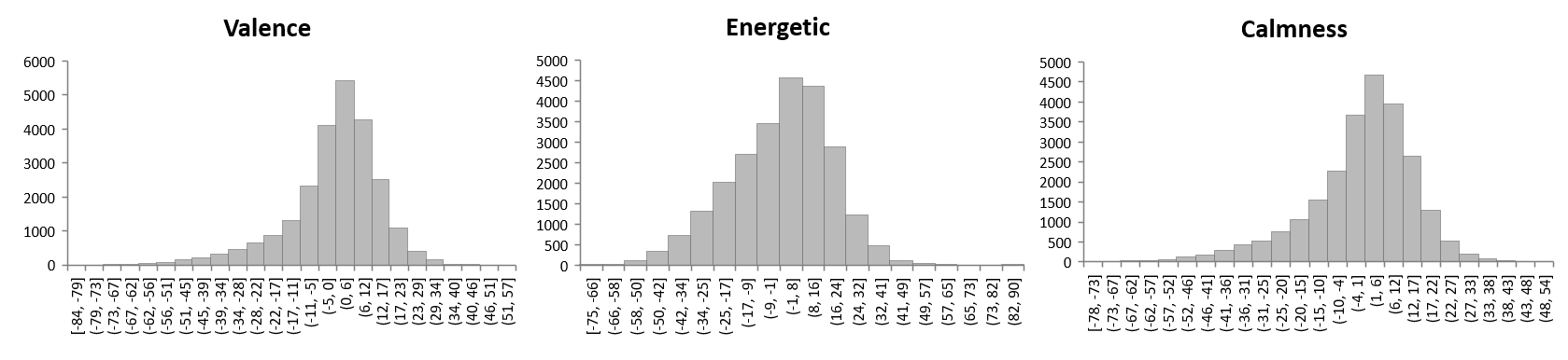
**

The histogram depicts the distribution (y-axis shows the frequency) of level-1 residuals (x-axis), which measure deviations from the conditional mean (conditional residuals) derived from our multi-level models (see Methods, section “Data analysis – ambulatory assessment”). Visual inspection confirmed that there was no serious deviation from normal distribution, indicating that our multi-level model is well suited to deal with the given data structure in this sample.

References

1. Glaesmer H, Schulz A, Hauser W, Freyberger HJ, Brahler E, Grabe HJ. The childhood trauma screener (CTS) - development and validation of cut-off-scores for classificatory diagnostics. Psychiatr Prax. 2013;40(4):220-6.

2. Bernstein DP, Fink L, Handelsman L, Foote J. Childhood trauma questionnaire. Assessment of family violence: A handbook for researchers and practitioners. 1998.

3. Agorastos A, Pittman JO, Angkaw AC, Nievergelt CM, Hansen CJ, Aversa LH, et al. The cumulative effect of different childhood trauma types on self-reported symptoms of adult male depression and PTSD, substance abuse and health-related quality of life in a large active-duty military cohort. J Psychiatr Res. 2014;58:46-54.

4. Lampert T, Kroll L, Müters S, Stolzenberg H. Measurement of the socioeconomic status within the German Health Update 2009 (GEDA). Bundesgesundheitsblatt, Gesundheitsforschung, Gesundheitsschutz. 2013;56(1):131-43.

5. Borkenau P, Ostendorf F. NEO-FFI : NEO-Fünf-Faktoren-Inventar nach Costa und McCrae. 2 ed. Göttingen: Hogrefe; 2008.

6. Laux L, Glanzmann P, Schaffner P, Spielberger CD. Das state-trait-angstinventar [The state-trait anxiety inventory]. Hogrefe, Göttingen (in German). 1981.

7. Döring N, Bortz J. Psychometrische Einsamkeitsforschung: Deutsche Neukonstruktion der UCLA Loneliness Scale. Diagnostica. 1993.

8. Traue HC, Hrabal V, Kosarz P. AlltagsBelastungsFragebogen (ABF): Zur inneren Konsistenz, Validierung und Stressdiagnostik mit dem deutschsprachigen daily stress inventory. Verhaltenstherapie und Verhaltensmedizin. 2000;21(1):15-38.

9. Jerusalem M, Schwarzer R. Self-efficacy as a resource factor in stress appraisal processes. Self-efficacy: Thought control of action. 1992;195213.

10. Hannöver W, Michael A, Meyer C, Rumpf HJ, Hapke U, John U. [Antonovsky's sense of coherence scale and presentation of a psychiatric diagnosis]. Psychother Psychosom Med Psychol. 2004;54(3-4):179-86.

11. Herzberg PY, Glaesmer H, Hoyer J. Separating optimism and pessimism: a robust psychometric analysis of the revised Life Orientation Test (LOT-R). Psychol Assess. 2006;18(4):433-8.

12. Topp CW, Østergaard SD, Søndergaard S, Bech P. The WHO-5 Well-Being Index: a systematic review of the literature. Psychother Psychosom. 2015;84(3):167-76.

13. Glaesmer H, Grande G, Braehler E, Roth M. " The German version of the Satisfaction with Life Scale (SWLS): Psychometric properties, validity, and population-based norms": Correction to Glaesmer et al.(2011). 2011.

14. Carver CS. You want to measure coping but your protocol's too long: consider the brief COPE. Int J Behav Med. 1997;4(1):92-100.

15. Schwarzer R, Schulz U. Soziale Unterstützung bei der Krankheitsbewältigung: Die Berliner Social Support Skalen (BSSS). Diagnostica. 2003;49(2):73-82.

16. Berhe O, Höflich A, Mößnang C, Reichert M, Kremer T, Gan G, et al. Reduced real-life affective well-being and amygdala habituation in unmedicated community individuals at risk for depression and anxiety. Biol Psychiatry Cogn Neurosci Neuroimaging. 2022.

17. Tost H, Reichert M, Braun U, Reinhard I, Peters R, Lautenbach S, et al. Neural correlates of individual differences in affective benefit of real-life urban green space exposure. Nat Neurosci. 2019;22(9):1389-93.

18. Reichert M, Braun U, Gan G, Reinhard I, Giurgiu M, Ma R, et al. A neural mechanism for affective well-being: Subgenual cingulate cortex mediates real-life effects of nonexercise activity on energy. Sci Adv. 2020;6(45).

19. Gan G, Ma R, Reichert M, Giurgiu M, Ebner-Priemer UW, Meyer-Lindenberg A, et al. Neural correlates of affective benefit from real-life social contact and implications for psychiatric resilience. JAMA psychiatry. 2021;78(7):790-2.

20. Wilhelm P, Schoebi D. Assessing mood in daily life: Structural validity, sensitivity to change, and reliability of a short-scale to measure three basic dimensions of mood. Eur J Psychol Assess. 2007;23(4):258-67.

21. Collip D, Oorschot M, Thewissen V, Van Os J, Bentall R, Myin-Germeys I. Social world interactions: how company connects to paranoia. Psychol Med. 2011;41(5):911-21.

22. Wichers M, Barge-Schaapveld DQ, Nicolson NA, Peeters F, de Vries M, Mengelers R, et al. Reduced stress-sensitivity or increased reward experience: the psychological mechanism of response to antidepressant medication. Neuropsychopharmacology. 2009;34(4):923-31.

23. Bolger N, Laurenceau J-P. Intensive longitudinal methods: An introduction to diary and experience sampling research. New York: The Guilford Press; 2013.

24. Reichert M, Tost H, Reinhard I, Schlotz W, Zipf A, Salize HJ, et al. Exercise versus Nonexercise Activity: E-diaries Unravel Distinct Effects on Mood. Med Sci Sports Exerc. 2017;49(4):763-73.
